# Exploring risky health behaviors and vulnerability to sexually transmitted diseases among transnational undocumented labor migrants from Bangladesh: a qualitative study

**DOI:** 10.1186/s12889-024-18696-3

**Published:** 2024-05-08

**Authors:** Md. Salman Sohel, Md. Khaled Sifullah, Babul Hossain, Md. Fouad Hossain Sarker, Noshin Tasnim Zaman, Md. Obaidullah

**Affiliations:** 1 Department of Development Studies, Daffodil International University, Dhaka-1216, Bangladesh; 2 Department of Nutrition and Food Engineering, Daffodil International University, Dhaka-1216, Bangladesh; 3 School of Humanities and Social Science, BRAC University, Dhaka-1212, Bangladesh; 4 Centre for Governance and Sustainability, Daffodil International University, Dhaka-1216, Bangladesh; 5 Department of Public Leadership, Management & Governance, East Delta University, Chattogram-4209, Bangladesh; 6 Global Migration Observer, Dhaka-1216, Bangladesh; 7 Centre For Global Migration Studies, Daffodil International University, Dhaka-1216, Bangladesh

**Keywords:** Irregular migration, Undocumented migration, Migrant worker, Risky health behaviors, HIV/STI risk, Bangladesh

## Abstract

**Background:**

In Bangladesh, remittances constitute a substantial portion of the country’s foreign exchange earnings and serve as a primary source of income. However, a considerable number of Bangladeshi citizens reside overseas without proper documentation, exposing them to significant challenges such as limited access to healthcare and socioeconomic opportunities. Moreover, their irregular migration status often results in engaging in risky health behaviors that further exacerbate their vulnerability. Hence, this study aimed to investigate the risky health behavior and HIV/STI susceptibility of Bangladeshi irregular international migrants residing across the globe with undocumented status.

**Methods:**

Using a qualitative Interpretative Phenomenological Approach (IPA), 25 illegal migrants were interviewed who are currently living illegally or returned to their home country. The author used a thematic approach to code and analyze the data, combining an integrated data-driven inductive approach with a deductive approach. Concurrent processing and coding were facilitated by employing the Granheim model in data analysis.

**Results:**

The study identified four risky health behaviors among irregular Bangladeshi migrants: hazardous living conditions, risky jobs, suicidal ideation, and tobacco consumption. Additionally, the authors found some HIV/STI risk behavior among them including engaging in unprotected sex, consuming alcohol and drugs during sexual activity, and having limited access to medical facilities.

**Conclusions:**

The findings of this study can be used by health professional, governments, policymakers, NGOs, and concerned agencies to develop welfare strategies and initiatives for vulnerable undocumented migrant workers.

## Introduction

The European Union (EU) defines irregular migrants as those who lack legal status in a host or transit country due to various reasons [1]. The term ‘illegal migrants’ remains controversial. To avoid this controversy, this study will use the term ‘undocumented migrants’.

Estimates indicate that there are approximately 50 million undocumented migrants worldwide, mainly residing in Western Europe and North America [2], while Europe had at least 3.9 million undocumented immigrants in 2017, possibly reaching up to 4.8 million [3]. Globally, undocumented migrants face striking challenges, including lack of access to healthcare, discrimination, exploitation, and abuse, leading to poor living conditions and health issues [4–7].

Migration has been a significant part of Bangladesh’s history. Bangladesh ranks as the 6th largest migrant sending and 8th largest remittance receiving country in the world [8]. Since 1976, Bangladeshi migrants have sent USD 235 billion in remittances home, with approximately 700,159 workers going abroad in 2019 to Gulf countries which alone attracted 647,000 Bangladeshi migrants [9, 10]. Undocumented labor migration is a significant global issue, but most studies have been conducted in a limited context. Research has focused on various aspects of irregular migration, including socioeconomic characteristics, security and social issues, exclusion, gender, trafficking, governance failures, and human development [4–6, 11]. Furthermore, several studies underscore the health challenges faced by undocumented migrants due to their restricted access to healthcare services [12–15].

Additionally, although a wealth of literature exists on HIV/STI risk behavior among migrant populations [6, 16–20], no study has specifically investigated the risky health behavior and HIV/STI risk factor of undocumented migrants. Furthermore, most studies have centered on the problems caused by undocumented migrants to the host country [21–31]. Even, Bangladeshi undocumented labour migrants are largely ignored for the difficulties they encounter in the host country.

This study addresses the lack of knowledge about the risky health behaviors and HIV/STI risk factors among irregular Bangladeshi international migrants. By identifying specific health behaviors and assessing the extent of risky behavior, the study’s findings will inform policymakers and stakeholders on effective health promotion strategies, protecting undocumented migrants, and preventing irregular migration. Additionally, the study may contribute to the literature on international migration, and health for vulnerable and marginalized populations. A proposed policy agenda outlines measures taken by various stakeholders to safeguard undocumented migrants and prevent irregular migration.

Hence, the research objective is to examine specific health behaviors exhibited by undocumented Bangladeshi international migrants and identify factors influencing HIV/STI risk among this population.

### Conceptual framework

The conceptual framework which guided the study “Exploring Risky Health Behaviors and Vulnerability to Sexually Transmitted Diseases Among Transnational Undocumented Labor Migrants from Bangladesh: A Qualitative Study” is below:

Illegal migrants are at risk of engaging in risky health behaviors. Studies have shown that migrants have high-risk sexual behavior and a low perception of HIV/STDs risk and healthcare needs [32]. Additionally, changes in health risk behaviors such as alcohol consumption, tobacco use, physical inactivity, and poor dietary habits have been observed among migrants with longer duration of residence [33]. Also, there is a high risk of disease transmission among migrants in northeastern Mexico, due to factors such as working to survive and fear of being traced [34]. Another study documented the emotional difficulties experienced by illegal Irish immigrants, including fear of deportation and limited freedom [35]. Castañeda (2009) highlighted disparities in healthcare access for unauthorized migrants in Germany, particularly in maternal and infant care, chronic illness management, and mental health support [36]. Policy, sociocultural, health, and sexual practice determinants: limited condom use, using drugs, no HIV test, multiple partnering, low HIV knowledge, and low perceived HIV risk, have been identified in previous research [37], while the influence of migration on HIV risk has also been highlighted [38]. Moreover, the high prevalence of STDs among female sex workers, particularly those who are transnational undocumented labor migrants, has been underscored [39]. Apostolopoulos (2006) further explores the specific risks faced by Mexican migrant laborers, including poverty, limited education, physical/social/cultural isolation, long work hours, hazardous work conditions, limited access to health care, low rates of condom use, multipartnering, and use of sexworkers [40].

The conceptual framework guiding the study is constructed upon existing literature and empirical evidence regarding the health risks and vulnerabilities faced by undocumented migrants, particularly in the context of risky health behaviors and HIV/STIs transmission. This framework is underpinned by various factors such as policy constraints, sociocultural determinants, and the influence of migration on health behavior. The analysis of these factors yields two central themes: risky health behavior and patterns of risky sexual behavior concerning HIV/STIs.

## Risky health behavior

This theme encompasses various dimensions of health risks and vulnerabilities experienced by transnational undocumented labor migrants, including:

- ***Hazardous Living Conditions***: Undocumented migrants often find themselves living in precarious and unsafe environments, which can exacerbate health risks and increase vulnerability to diseases [41, 42].
- ***Suicidal Ideation***: The psychological distress associated with undocumented status, social isolation, and economic hardship may lead to suicidal ideation among migrants [43–45]. Suicidal ideations (SI), commonly referred to as thoughts or ideas of suicide, encompass a wide spectrum of considerations, desires, and fixations concerning death and self-harm.
- ***Risky Job***: Undocumented Migrants frequently engage in employment characterized by hazardous conditions, long hours, and limited access to healthcare, amplifying their susceptibility to health problems [46, 47].
- ***Tobacco Consumption***: There is a prevalence of tobacco use among undocumented migrants, which contributes to their overall health risks and exacerbates existing health conditions [48, 49].

## Risky sexual behavior patterns 

This theme focuses on the specific patterns of risky sexual behavior observed among transnational undocumented labor migrants, including:

- ***Unprotected Sex***: Migrants often engage in unprotected sexual activities, increasing their vulnerability to HIV/STIs transmission [50, 51].
- ***Using Drugs and Alcohol During Sex***: Substance use during sexual encounters is common among undocumented migrants, which heightens their risk of engaging in risky behaviors and contacting HIV/STIs [52–54].
- ***No Medical Check-up***: Due to various barriers, including fear of deportation and limited access to healthcare services, undocumented migrants frequently forego regular medical check-ups, further exacerbating their vulnerability to HIV/STIs [55, 56].

The conceptual framework underscores the multifaceted nature of health risks and vulnerabilities faced by transnational undocumented labor migrants, highlighting the interplay between socio-political factors, migration dynamics, and individual health behaviors. By elucidating these themes and sub-themes, the study aims to provide insights into the complex factors shaping the health outcomes of undocumented migrants and inform targeted interventions aimed at mitigating their health risks and promoting well-being.

## Materials and methods

### Research approach and design

When selecting the research strategy for this study, various factors were taken into consideration. The study aimed to investigate the risky health behavior and HIV/STI risk factors of Bangladeshi undocumented migrants using a qualitative phenomenological framework. The qualitative phenomenology research approach enabled the exploration and observation of phenomena from the participants’ perspectives. Qualitative phenomenology is concerned with how individuals understand and perceive their experiences and environment [57]. To gain insights into the participants’ lived experiences, the Interpretative Phenomenological Approach (IPA) developed by Smith (1996) and Smith & Osborn (2015) was utilized [58]. Chapman & Smith (2002) argue that lived experiences should be understood primarily through the lens of the participant’s experiences rather than preconceptions based on theoretical assumptions [59].

### Sample size

Qualitative investigations typically involve fewer samples than quantitative analyses to obtain statistical and numerical results [60]. Researchers have provided additional guidance on selecting sample sizes for qualitative research. Researchers can improve open and thoughtful communication by working with fewer than twenty participants; building and maintaining close relationships [61]. For different types of qualitative research, 15 to 20 interviewees are considered optimal. Qualitative research should involve a minimum of 20 participants. In this study, a non-probability purposive sampling technique was used to select respondents [62]. We conducted 25 in-depth interviews with undocumented Bangladeshi migrants who had worked illegally in five countries: The Kingdom of Saudi Arabia, Iraq, Malaysia, Libya, and Italy. Five sets of in-depth interviews were collected from each country. The main characteristics of the study participants are as listed on Table 1.

**Table 1:** Demographic information of study participants

Participant		Age	Education	Marital Status	Years of Experience	Transnational Labour Migration Experience	Current Status	Prison lived in Host Country
1	42		9 grades	Married	10	KSA	Returnee	Yes
2	27		3 grades	Unmarried	2	KSA	Non-Returnee	No
3	30		8 grades	Unmarried	5	KSA	Returnee	Yes
4	39		10 Class	Married	10	KSA	Returnee	Yes
5	30		5 grades	Unmarried	6	KSA	Returnee	Yes
6	46		illiterate	Married	11	Italy	Non-Returnee	No
7	34		Illiterate	Married	6	Italy	Non-Returnee	No
8	32		3 grades	Married	5	Italy	Returnee	No
9	41		7 grades	Divorcee	9	Italy	Non-Returnee	Yes
10	27		Illiterate	Unmarried	4	Italy	Returnee	Yes
11	44		8 Class	Married	10	Malaysia	Returnee	No
12	34		10 grades	Married	5	Malaysia	Returnee	No
13	25		9 grades	Married	4	Malaysia	Non -Returnee	Yes
14	40		12 grades	Unmarried	6	Malaysia	Returnee	No
15	23		2 grades	Unmarried	2	Malaysia	Non-Returnee	No
16	24		8 grades	Married	3	Libya	Non-Returnee	Yes
17	53		5 grades	Married	12	Libya	Returnee	No
18	36		Bachelor	Married	5	Libya	Returnee	Yes
19	31		5 grades	Married	3	Libya	Non-Returnee	No
20	25		10th grade	Married	2	Libya	Non-Returnee	No
21	35		8 grades	Unmarried	7	Iraq	Returnee	No
22	28		7 grades	Married	5	Iraq	Returnee	Yes
23	30		12 grades	Married	4	Iraq	Returnee	Yes
24	35		9 grades	Married	7	Iraq	Non-Returnee	No
25	29		4 grades	Married	4	Iraq	Returnee	Yes

### Data collection procedure, instruments

The study’s subject matter was explored through semi-structured interviews with participants [63]. Semi-structured interviews, as explained by Berg (2012), enable a more in-depth examination of research questions. A semi-structured interview was conducted to gain a deeper understanding of the participants’ perspectives [64]. Data were collected from 15 July 2022 to 15 December 2022. The first step in data collection was to gather three interview samples from participants, prepare and develop the questionnaire, and tailor the questions to suit the interview context. In qualitative studies, questionnaires are usually developed through an iterative process that builds upon the original interviews. For this study, we collected a total of 25 in-depth interviews. We conducted face-to-face interviews with 15 undocumented migrants who had worked illegally in Saudi Arabia, Iraq, Malaysia, Libya, and Italy. Furthermore, we conducted ten in-depth online interviews with individuals who were currently working illegally in the aforementioned countries. The data were collected from various locations within the Jessore district.

To conduct the online interviews, we used WhatsApp, Zoom Meeting, and Google Meet. The interviews ranged in duration from 52 to 118 min and were recorded using various devices. Some respondents were hesitant to speak while being recorded, and consequently, the interviews were written. We closely observed the interviewees’ attitudes, expressions, tone, and body language throughout the interviews. After the interviews were conducted, our research assistants carefully verified the transcripts to ensure the accuracy of the information.

### Quality assurance: data analysis technique, validity, and reliability

An indispensable aspect of qualitative data analysis using NVivo software is its excellent analytical tools [65, 66] that facilitates coding, categorization, and theme creation [67, 68]; it provide a paperless and efficient way to manage and analyze data. To ensure the validity and reliability of the findings, NVivo-12 was used to meticulously code, classify, and structure interview transcripts. Also, a triangulation approach was employed in the data collection process to avoid bias and enhance the data’s quality [69, 70]. A multi-researcher team, including myself and research assistants, collected the data by conducting field investigations regularly, adhering to investigator triangulation norms, and employing a meticulous data collection and processing approach.

### Approaches for measuring and coding data

The author utilized a thematic approach for coding the data and performed data analysis through a hybrid of integrated data-driven inductive approach [71] and deductive approach [72]. For concurrent processing and coding, the Granheim model [73] was employed in the data analysis. Thematic analysis approach is illustrated in Table 2.

**Table 2:** Qualitative data analysis using Granheim approach

Steps	Description
1. **Interview transcription**	The interviews were taped and read again after hearing the recordings several times to comprehend the contents.
2. **Unit for the formation of** **meaning analysis**	All interviews were analyzed as a single unit. Creating primary codes by abstracting meaning units
3. **Comprehensive sorting of** **similar codes**	The grouping of similar fundamental codes into more comprehensive categories.
4. **Comparison of codes and establishment of subcategories**	In contrast, all codes and data identified similarities and differences. This process resulted in the formation of categories and subcategories.
5. **Comparing subcategories and establishing primary categories**	The initial interviews yielded a set of codes, categories, and subcategories, and the emerging codes were considered the results due to the content analysis methodology. Two independent researchers examined category data.

## Results

The study involved 25 participants whose marital status was divided into 60% married and 40% unmarried. These individuals hailed from various countries due to transnational labor migration, with each country contributing 20% of the sample size. The countries represented included Saudi Arabia (KSA), Italy, Malaysia, Libya, and Iraq. Regarding their current status, 44% were returnees while 56% were non-returnees. Additionally, 44% of the participants reported having lived in a prison in the host country, while the remaining 56% had not. On average, participants had approximately 5.68 years of experience in their respective fields, with an average age of around 31.72 years (Table 3).

**Table 3:** Quantitative characteristics of the respondents

Marital Status (*n* = 25)	Country (Transnational Labour Migration) (*n* = 25)	Current Status (*n* = 25)	Prison lived in Host Country (*n* = 25)	Average years of experience	Average age
Married = 60% Unmarried = 40%	• KSA20% • Italy20% • Malaysia 20% • Libya 20% • Iraq20%	Returnee = 44% Non-Returnee = 56%	Yes = 44% No = 56%	5.68 years	≈ 31.72 years

**Fig. 1:**
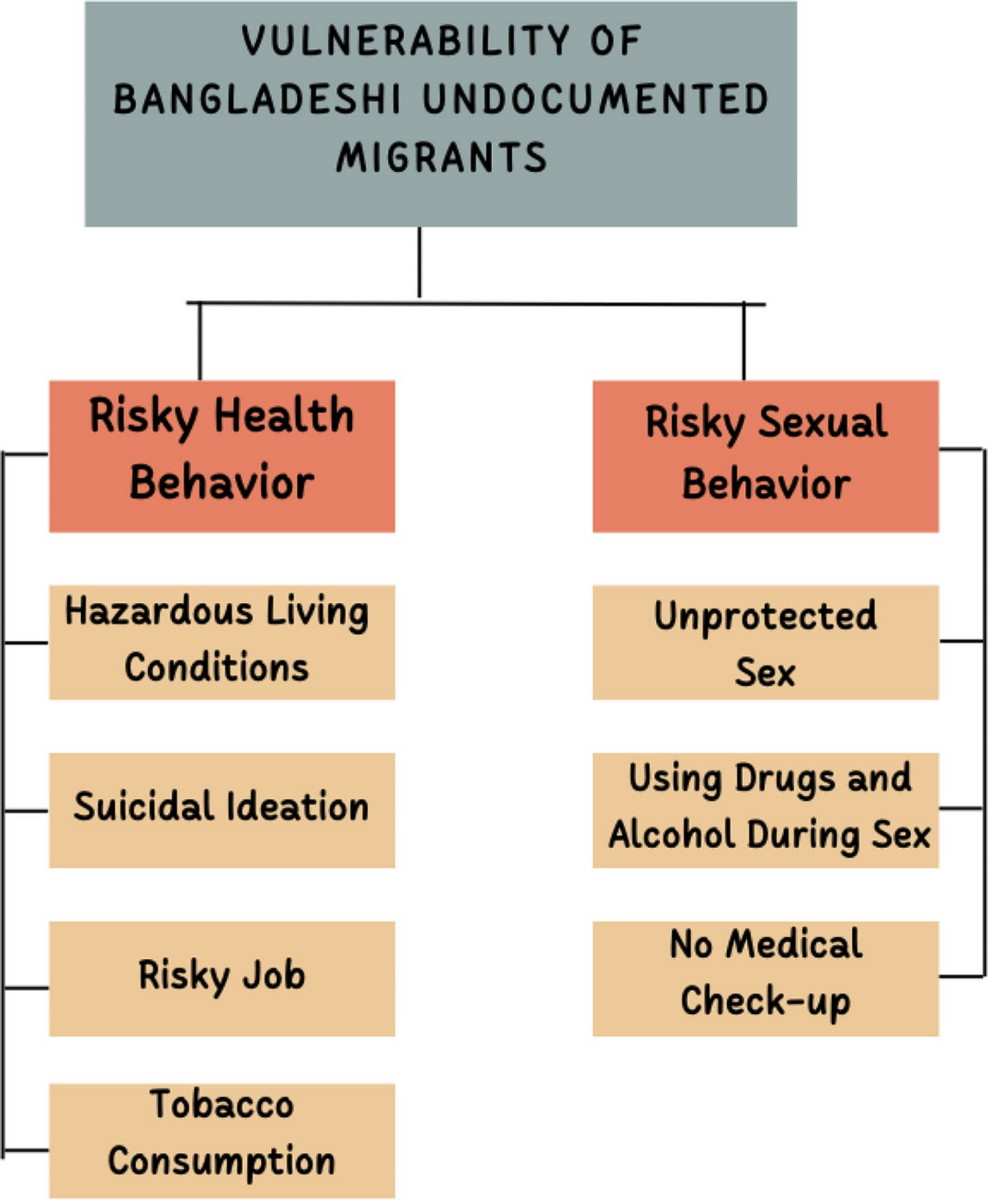
Conceptual framework of the Study

This section presents the primary findings of the study, which were derived through qualitative data analysis utilizing NVivo-12 software and guided by our conceptual framework (Fig. 1).

### Risky health behavior

While all migrants may encounter obstacles in accessing healthcare, undocumented migrants are particularly susceptible to specific hazards and illnesses. Our findings indicate that undocumented migrants exhibit highly risky health behaviors.

#### Hazardous living conditions

Migrants are more susceptible to income and health-related risks than native-born workers. Undocumented migrants, in particular, often reside in substandard and isolated conditions. Our study’s 13 respondents disclosed that they do not sleep inside their living quarters because of the risk of being caught during a police raid. Instead, they seek out locations where they can quickly flee if law enforcement arrives, such as the jungle, open areas, or the back of their dwelling.

As one 32-year-old migrant (R #15) stated, *“I am constantly fearful of the police because of my illegal migrant status. As a result, I have difficulty sleeping at night and often seek refuge in a small hut at the back of the building, which leaves me vulnerable to mosquito bites.“*

Annually, thousands of individuals become transnational migrants, with around half being workers. These workers often perform jobs that endanger their health, particularly if they are undocumented, and their living conditions can be uncomfortable. Compared to non-migrant workers, they typically work longer hours for less pay in worse conditions and are often subjected to human rights violations, abuse, and violence. Adverse occupational exposures and working conditions are more prevalent among migrant workers worldwide, leading to poor health outcomes, workplace injuries, and occupational fatalities. In this instance, Respondent #1 recounted, *“The company does not provide us with adequate accommodation; we have ten people living in a small room, and ten people share a bathroom for bathing and using the toilet, which frequently causes problems.”*

#### Suicidal ideation

Suicidal ideation can stem from various underlying factors. It often occurs when an individual is confronted with overwhelming circumstances that exceed their ability to cope. The study identified specific reasons why undocumented Bangladeshi migrants may experience suicidal ideation. Seven respondents reported that their undocumented status prevented them from finding employment despite paying substantial amounts to migrate, resulting in financial instability and extreme food scarcity that gradually affected their health and well-being. Due to the risk of being caught by the police, they were unable to sleep properly and often resorted to sleeping in open places or the jungle. These unbearable living conditions led them to contemplate suicidal thoughts.

One respondent, a 57-year-old migrant identified as R #14, shared, *“My visa has expired, and obtaining new paperwork will cost a lot of money. Additionally, I am unable to find work without legal papers, and I am unable to send money back home. I cannot sleep at night or eat three meals a day, and sometimes I think it would be better to die than to continue living this difficult life.”*

#### Risky job

Each year, hundreds of foreign employees suffer injuries or lose their lives in preventable workplace accidents. The study revealed that undocumented workers, particularly those employed as labor contractors in Malaysia, lack legal protections. These workers reported being subjected to poor and unsafe working conditions, with inadequate protective gear and training. Respondent #18 recounted, *“The company forces us to do the hardest work because we are illegal. One of my acquaintances fell to his death while painting or constructing a building last year.“*

Several participants shared that they were compelled to work in hazardous environments, resulting in minor injuries, equipment damage, and in some cases, serious harm or fatalities. It is crucial for employees to remain vigilant and aware of their surroundings to prevent mishaps, as anything can happen.

Respondent #7 shared their experience, *“I worked for a company that manufactured steel and rods. I had to work near flames that reached temperatures of around 200 degrees Celsius. The heat was so intense that it could be felt from ten to twelve hands away. I had no choice but to go and burn the chains. This was extremely dangerous, and two other people died because of it.”*

#### Tobacco consumption

Our study revealed that tobacco consumption is a prevalent habit among irregular migrants, with most participants reporting being smokers. When asked why they smoke, respondent #13 shared, *“I suffer from depression and often feel lonely. Smoking helps to alleviate my depressive symptoms.“*

In our study, we found that most illegal migrant workers spend their days in anxiety. It is an important risk factor for irregular migrant workers. Regular smoking is a cause of cardiovascular diseases and premature death. We tried to know deeply why they take tobacco. Respondent#13 stated that.

*Q: Why do you take tobacco?*

*A: Being away from family is very difficult. Here, I have to go through a difficult situation. I am an illegal worker without papers and always run away to avoid being caught by the police. I smoke regularly to forget them.*

*Q: Can smoking reduce suffering?*

*A: When I don’t smoke, I feel depressed and have sleeping difficulty. That’s because I do this knock-out anxiety.*

### HIV/STD risk behavior

Migrants are exposed to several risk factors that influence HIV and STD susceptibility and vulnerability patterns in populations affecting HIV/STD transmission. Due to inadequate access to good health care services, protection, justice, precarious housing, and job situations, undocumented migrants are at increased risk of getting HIV [74]

#### Unprotected sex

Although sex is a natural and healthy aspect of life, it can be dangerous if behaviors that transmit diseases or cause physical or mental harm are involved. Unprotected sex poses a significant risk of contracting HIV and other sexually transmitted infections (STIs), as bodily fluids such as blood and semen are exchanged during intercourse. Our interviews found that most respondents engaged in unprotected sex due to various reasons. They perceived condoms as expensive and feared being caught by the police when buying them due to their undocumented status. Additionally, some respondents believed that sex without a condom provided greater pleasure, ignoring the importance of condom use during sexual activity.

Respondent #9, a 50-year-old migrant, stated, *“I was hesitant to purchase condoms due to my illegal status, as I feared being caught by the police. Furthermore, I did not want to waste money on buying condoms. I preferred having sex without a condom, as it was more enjoyable.“*

To get HIV or another STD when someone has more than one sex partner or many sex partners during someone’s lifetime. More people mean more chances that one or more of them will have HIV or an infection. After taking the interview with all our respondents, we found that many of the undocumented migrants were addicted to having sex with multiple sex partners or having sex with many call girls in their expatriate lives. In this case, **Respondent #3;** stated that.

We used to have 8–10 people together in the same building. We hired the call girl in our room 1–2 times a month and had sex together. What to do? We have to meet our physical needs, addressing them as a brother. We couldn’t go anywhere without Call Girl as we were illegal.

The study also revealed that some undocumented migrants enjoyed their sexual activity around the anal area of their female partner, which they learned from pornography videos or movies. It is the sex that poses the greatest risk to both men and women of contracting and spreading STDs like HIV. The lining of the anus is much thinner than the vagina so it can be damaged much more quickly. This greatly increases its susceptibility to infection.

Having sex on the back makes me feel better than on the front. So, when I hired a girl, I must be clear to tell her before that I should be allowed to use the back side, but I had to pay more for it. (R#11, 39 years old migrants).

#### Using drugs and alcohol during sex

Someone can easily get infected by HIV and other diseases, including hepatitis from someone who does sex with drugs injected person. Moreover, if the person you’re having sex with shares drug equipment with someone HIV positive, they can contract the virus. Usually, migrants mitigate their sexual demands abroad with a prostitute. These prostitutes are used to involve taking drugs; even though they have to spend very intimate time with different types of people in their profession, they may be drug-addicted. As a result, when undocumented Bangladeshi migrants do sexual activities without thinking about safe sex, they fall into danger that they don’t know. In this favor, one respondent disclosed that:

When I went to have sex with my partner, she said wait; then I saw he took Yaba started eating with marijuana and cigarette and told me to eat it. After doing these, if you have sex, you will enjoy it for a long time. (R #13, 21 years old migrants).

Based on our Nvivo analysis, the study explored that most of their sexual time they and their partner used to take any type of alcohol like wine, etc., so they have no real sense when they take it. In this case, they fall into unprotected sex. Even though they don’t separate what is wrong or right, it leads to being less careful. Re #10; expressed like.

When we drink too much alcohol or Yaba, we have no sense of what happens. Once after having sex, I noticed that I was asleep; my partner had left with my mobile and wallet. And will you remember to use condoms after eating these?

#### No medical check-up

Irregular migrants live at least 5 to 10 years in their destination state. We found that they do sex without a condom. On the other hand, they are not allowed in healthcare services. So, they can’t do the medical screening. Even some participants acknowledged they have sexually transmitted diseases. But they don’t go to the hospital for better treatment as they are not allowed to. So, we think they are at a high risk of receiving the country and sending the country. In this regard, respondents #3#21, #25#17 said.

I found I have some sexual diseases like syphilis. I went to the local clinic, but they didn’t give me any treatment because I don`t have a visa on my passport. I am taking self-treatment now at home.

## Discussion

This study explored that irregular migrants have risky health behavior. Similarly, to that [75], said undocumented migrants are viewed as posing a greater health risk. Due to their irregular status and the implications of economic and social marginalization, undocumented migrants are at a greater risk for health issues [76]. Migrants who engage in sexual activities are at risk of contracting and spreading HIV not only in their country of residence but also in their country of origin when they visit relatives. As a result, these travelers can serve as a means for the cross-border transmission of sexually transmitted infections, including HIV [77].

Undocumented migrants often live in unsanitary conditions and avoid detection by law enforcement by seeking out safe places, which can expose them to mosquito-borne illnesses like dengue and malaria. They may also avoid sleeping indoors for fear of being caught in police raids. Undocumented migrants face longer work hours, lower pay, and worse working conditions than non-migrants, and they are also at risk of violence and human rights violations. This is a global issue, as migrant workers worldwide are exposed to unfavorable working conditions that can lead to negative health outcomes and occupational accidents [78].

Besides, suicide is a serious issue for social welfare and public health around the world. Asia has a higher suicide rate than the rest of the world [79] [80]. discussed that Nepalese migrant laborers have numerous difficulties in South Korea, the study recognized and ranked eight sources of distress and perceived suicide risks, both at home and in the host nation. A wide range of socio-cultural, behavioral, occupational, physical, and mental health problems as well as communication hurdles are among the perceived risks for suicide. Based on our research, several factors such as lack of proper documentation, substandard living conditions, and inability to send money home, can significantly contribute to individuals attempting suicide.

Over half of transnational migrant workers frequently perform hazardous jobs. Most significantly, these precarious workers might take more risks while at work, do their duties without the proper training or safety gear, and do not voice their concerns about hazardous working circumstances [81]. Our study revealed that undocumented workers in Malaysia, who primarily serve as laborers, lack legal rights and protections. Participants reported frequent exposure to hazardous and unhealthy working conditions, often without receiving proper training or access to protective equipment.

Irregular migrants are more susceptible to HIV/AIDS since they don’t use condoms [82]. Low condom use, multiple partners, and early sexual exploration are risky behavior among young migrants they believed they were in danger of contracting an STI or developing HIV/AIDS by engaging in high-risk behavior [83]. In our study, we found that a substantial proportion of respondents preferred having unprotected sex. This was mainly attributed to the perception that sex without a condom is more pleasurable than with a condom, and that purchasing protection can be costly and risky for undocumented migrants. As a result, they often engage in sexual activity without considering the potential consequences of unprotected sex.

 [84] analyzed that there was a relationship between drug use and HIV risk among migrant female sex workers in the US Virgin Islands. HIV transmission is influenced by a variety of circumstances, including drug use, migration, and commercial sex. Our study identified that undocumented migrants face an increased risk of harm when they engage in sexual activity while using drugs and alcohol. Substance use can lead to loss of consciousness, and as a result, undocumented Bangladeshi migrants engaging in sexual activity without considering safe sex are at risk of harm.

Foreign workers are usually deprived of from taking health care services [85]. Undocumented migrants in Denmark reported having trouble getting medical care. Fear of police reporting limited medical rights, healthcare workers’ arbitrary attitudes, and low language skills are the reasons for irregular migrants to take service from healthcare [81, 86]. Findings from our study indicate that Bangladeshi workers who migrated internationally stated unequivocally that undocumented (illegal) migrant laborers are at a higher risk of contracting HIV due to limited access to medical care and health information, as compared to regular migrants. These workers are denied medical assistance and health protection, and their fear of hospitals and clinics further exacerbates their lack of access to medical care. Access to medical care is frequently limited for many undocumented workers. A prior study in Bangladesh found that marginalized populations tend to avoid seeking formal healthcare and instead frequently visit drug shops [87].

## Conclusion and policy recommendations

Irregular migration is a highly complex and sensitive issue that requires careful governance at both local and international levels. According to the International Organization for Migration, an estimated fifty million people worldwide engage in irregular migration (ILO, 2022). Undocumented migrants residing in host countries are known to engage in highly risky health behaviors, including living in hazardous conditions, having suicidal thoughts, working in unsafe jobs, and consuming tobacco.

Undocumented Bangladeshi migrants are particularly vulnerable to HIV and STDs due to various risk factors, which may have implications for both the host and sending countries. These risk factors include engaging in unprotected sexual activity, using drugs and alcohol during sex, and lacking access to health services due to their undocumented status. Addressing these challenges is crucial to reduce the transmission of HIV/STDs and improve the health outcomes of undocumented migrants in both the host and sending countries.

Recommendations based on the research findings are as follows:

1. Conduct a thorough analysis of the root causes of irregular migration and streamline the bureaucratic procedures involved in legal migration.
2. Explore new labor markets to reduce irregular migration and maintain regional cooperation for effective migration control.
3. Establish mobile clinics or health centers to improve healthcare access for undocumented migrants and provide free or low-cost medical care, including HIV/STD testing, treatment, and counseling.
4. Collaborate with NGOs and other stakeholders to raise awareness about the dangers of irregular migration and unsafe sexual practices, as well as provide education on HIV/STD prevention and promote safe sexual practices.
5. Enforce labor laws that protect the rights of all workers, including undocumented migrants, by taking measures to prevent exploitation, improve working conditions, and ensure fair wages.

## Data Availability

All data generated or analyzed during this study are included in this published article.
